# Synthesis, crystal structure and thermal properties of poly[bis­[μ-3-(amino­meth­yl)pyridine-κ^2^ *N*:*N*′]bis(thio­cyanato-κ*N*)manganese(II)]

**DOI:** 10.1107/S2056989021006733

**Published:** 2021-07-02

**Authors:** Christoph Krebs, Inke Jess, Christian Näther

**Affiliations:** a Institut für Anorganische Chemie, Christian-Albrechts-Universität zu Kiel, Max-Eyth-Str. 2, D-24118 Kiel, Germany

**Keywords:** crystal structure, layered structure, thermal properties, manganese thio­cyanate, 3-(amino­meth­yl)pyridine, isotypism

## Abstract

The reaction of Mn(NCS)_2_ with a stoichiometric amount of 3-(amino­meth­yl)pyridine in ethanol led to the formation of the title compound, [Mn(NCS)_2_(C_6_H_8_N_2_)_2_]_*n*_, which is isotypic to its Zn, Co and Cd analogues. The manganese cation is located on a centre of inversion and is octa­hedrally coordinated in an all-*trans* configuration by two terminal N-bonded thio­cyanate anions as well as four 3-(amino­meth­yl)pyridine co-ligands, of which two coordinate with the pyridine N atom and two with the amino N atom. The 3-(amino­meth­yl)pyridine co-ligands connect the Mn^II^ cations into layers extending parallel to (10  ). These layers are further connected into a three-dimensional network by relatively strong inter­molecular N—H⋯S hydrogen bonding. Comparison of the experimental X-ray powder diffraction pattern with the calculated pattern on the basis of single-crystal data proves the formation of a pure crystalline phase. IR measurements showed the CN stretching vibration of the thio­cyanate anions at 2067 cm^−1^, which is in agreement with the presence of terminally N-bonded anionic ligands. TG–DTA measurements revealed that the title compound decomposes at about 500 K.

## Abstract 2

In the crystal structure of the title compound, the manganese cations are octa­hedrally coordinated by the N atoms of four 3-(amino­meth­yl)pyridine co-ligands and two terminal N-bonded thio­cyanate anions. The metal cations are linked into layers by the co-ligands, and these are further connected into a three-dimensional network by inter­molecular N—H⋯S hydrogen bonding.

## Chemical context   

In contrast to other small-sized ligands such as azide or cyanide anions, thio­cyanate anions show many more coordination modes. Therefore, a variety of structures including discrete complexes (Prananto *et al.*, 2017 ▸; Małecki *et al.*, 2011 ▸; Wöhlert *et al.*, 2014 ▸), dimers (Mautner *et al.*, 2015 ▸; Wei & Luo, 2010 ▸; Jochim *et al.*, 2018 ▸), chains (Mautner *et al.*, 2018 ▸; Rams *et al.*, 2020 ▸), layers (Suckert *et al.*, 2016 ▸, 2017 ▸) or in very rare cases three-dimensional networks (Suckert *et al.*, 2017 ▸) can be observed. This structural variability is further enhanced by isomerism, because for an octa­hedral coordination with three pairs of identical ligands, five different isomers exist, including the all-*trans*, all-*cis* and three different *cis*-*cis*-*trans* configurations. These features are found in compounds with structures where the metal cations are linked by pairs of anionic ligands into chains. The majority of compounds with *μ*-1,3-bridging thio­cyanate anions shows this behaviour. Depending on the actual metal coordination (all-*trans* or *cis*-*cis*-*trans*), linear or corrugated chains are observed (Jin *et al.*, 2007 ▸; Rams *et al.*, 2017 ▸; Böhme *et al.*, 2020 ▸; Jochim *et al.*, 2020 ▸). Moreover, even for compounds with layered thio­cyanate structures, different networks are realized, in which the metal cations are linked exclusively by single anionic ligands or by both singly and doubly *μ*-1,3-bridging thio­cyanate anions. For less chalcophilic metal cations like Mn^II^, Fe^II^, Co^II^ or Ni^II^, the majority of compounds consist of structures with only terminally N-bonding thio­cyanate anions, because in this case this coordin­­ation is energetically favoured. With only mono-coordinating ligands this usually leads to the formation of discrete metal complexes with an octa­hedral coordination. If bridging co-ligands are used, chain structures can be realized and networks of higher dimensionality are available if additional *μ*-1,3-bridging thio­cyanate anions are present. 
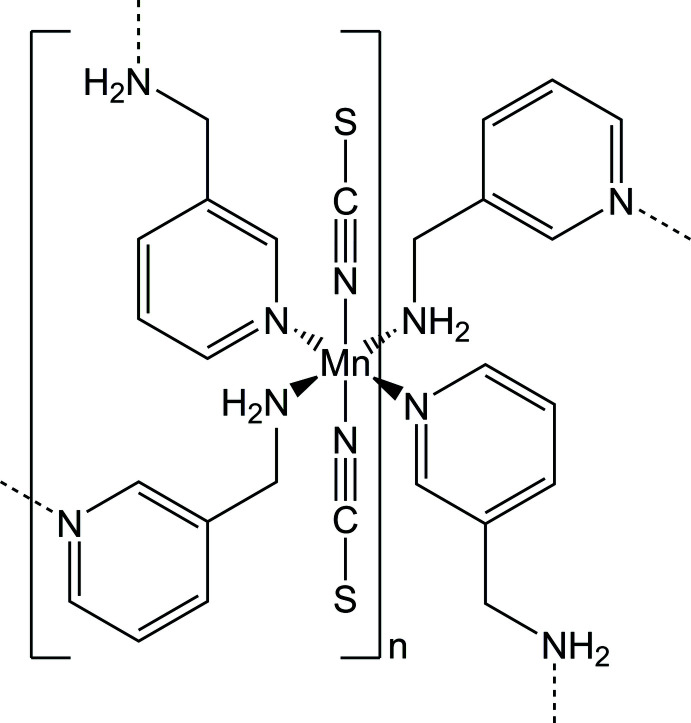


Thio­cyanate coordination polymers are of inter­est not only because of their variable structural behaviour, but also because this ligand is able to mediate reasonable magnetic exchange. We and other groups have reported many new compounds in which the metal cations are linked by *μ*-1,3-bridging thio­cyanate anions into chains or layers (Werner *et al.*, 2015 ▸; Bassey *et al.*, 2020 ▸; Mekuimemba *et al.*, 2018 ▸; Palion-Gazda *et al.*, 2015 ▸; Neumann *et al.*, 2019 ▸; Mousavi *et al.*, 2020 ▸). In this context, we became inter­ested in 3-(amino­meth­yl)pyridine, because this ligand is able to link metal cations *via* the pyridine and the amino N atom. Surprisingly, with Co^II^ we always obtained only one crystalline phase in which the Co^II^ cations are coordinated by only terminally N-bonding thio­cyanate anions but linked into layers by the 3-(amino­meth­yl)pyridine co-ligands (Krebs *et al.*, 2021 ▸). In contrast to the Co^II^ cation, the Mn^II^ cation is more chalcophilic and usually behaves like Cd^II^, for which compounds with *μ*-1,3-bridging thio­cyanate anions are much easier to obtain. Therefore, we used Mn(NCS)_2_ in the present study. However, irrespective of the ratio between Mn(NCS)_2_ and 3-(amino­meth­yl)pyridine, we always obtained only one crystalline phase with compos­ition Mn(NCS)_2_(C_6_H_8_N_2_)_2_. Single-crystal structure analysis revealed isotypism with the Co^II^ analogue reported recently (Krebs *et al.* 2021 ▸). Comparison of the experimental X-ray powder diffraction pattern with the calculated pattern based on single-crystal data proved that a pure crystalline phase was obtained (see Fig. S1 in the supporting information); IR investigations revealed that the CN stretching vibration is observed at 2067 cm^−1^, in agreement with the presence of only terminally N-bound thio­cyanate anions (Fig. S2). TG–DTA measurements showed decomposition of the compound at about 500 K, which is accompanied by an endothermic event in the DTA curve (Fig. S3). The first decomposition step might be associated with the removal of the 3-(amino­meth­yl)pyridine co-ligand. On further heating, an exothermic signal is observed, which indicates the decomposition of the co-ligand.

## Structural commentary   

**Figure 1:**
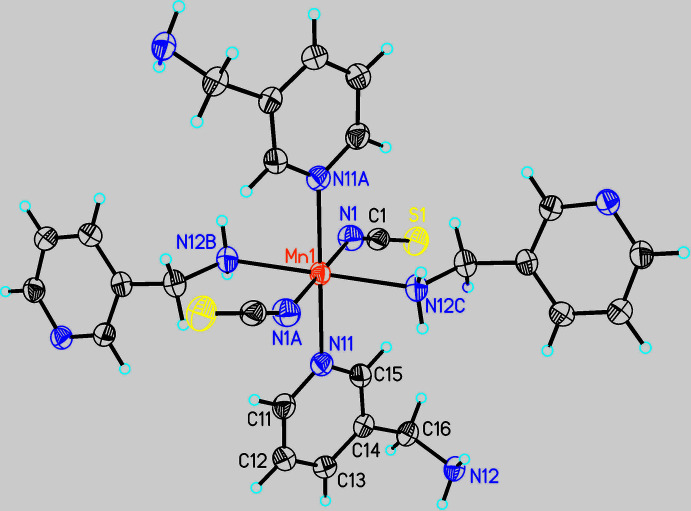
The coordination of the Mn^II^ cation in the title compound with displacement ellipsoids drawn at the 50% probability level. [Symmetry codes: (A) −*x*, −*y* + 1, −*z*, (B) 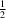 − *x*, − 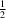 + *y*, 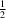 − *z*, (C) − 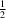 + *x*, 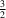 − *y*, − 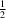 + *z*.]

**Figure 2:**
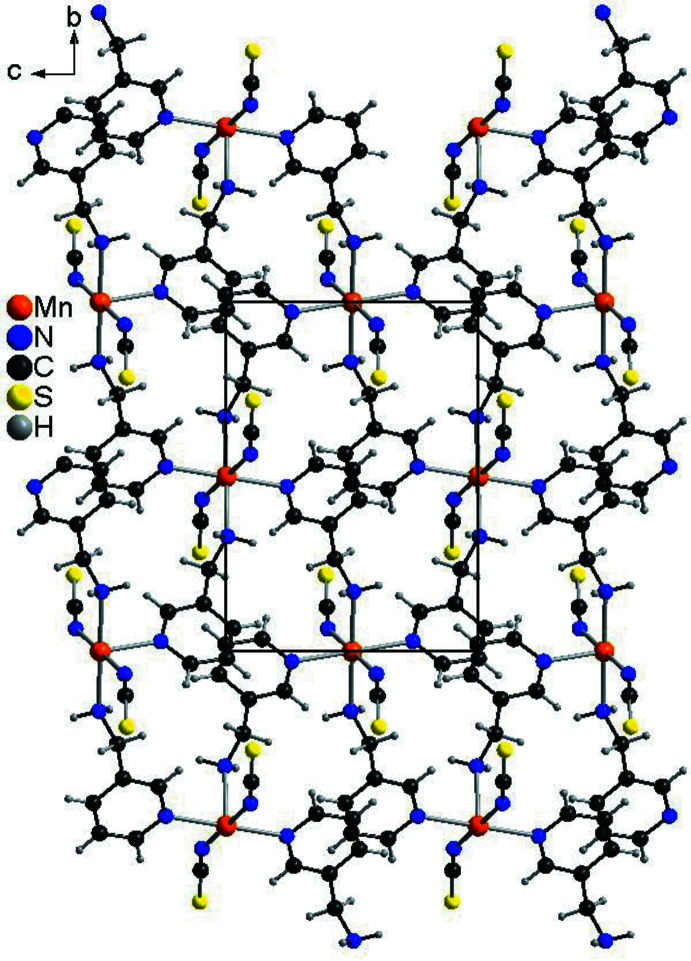
Crystal structure of the title compound in a view of a layer along the crystallographic *a* axis.

Mn(NCS)_2_(C_6_H_8_N_2_)_2_ is isotypic with its recently reported Cd^II^, Zn^II^ and Co^II^ analogues (Neumann *et al.*, 2017 ▸; Krebs *et al.*, 2021 ▸). The asymmetric unit consists of one Mn^II^ cation that is located on a centre of inversion as well as one 3-(amino­meth­yl)pyridine co-ligand and one thio­cyanate anion (Fig. 1 ▸). The Mn^II^ cation is octa­hedrally coordinated by the N atoms of four symmetry-equivalent 3-(amino­meth­yl)pyridine co-ligands and two symmetry-equivalent thio­cyanate anions. Two of these co-ligands coordinate through the pyridine N atom whereas the other two coordinate with the amino N atom. Each pair of identical donor atoms is in a *trans*-position (Fig. 1 ▸). The Mn—N bond length to the negatively charged thio­cyanate N atom is significantly shorter than that to the 3-(amino­meth­yl)pyridine co-ligand; the Mn—N bond length to the pyridine N atom is significantly longer than that to the amino N atom of the 3-(amino­meth­yl)pyridine ligand (Table 1 ▸). As expected, all Mn—N bond lengths are significantly longer and shorter, respectively, compared to the Co^II^ and Cd^II^ analogues. The bond angles around Mn^II^ indicate a considerable distortion (Table 1 ▸). This is also indicated by the mean octa­hedral quadratic elongation of 1.0013 and the octa­hedral angle variance of 0.8258 (Robinson *et al.*, 1971 ▸). The Mn^II^ cations are connected by bridging 3-(amino­meth­yl)pyridine ligands into chains, which are further linked into layers extending parallel to (10 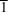 ) by additional co-ligands (Fig. 2 ▸).

## Supra­molecular features   

**Figure 3:**
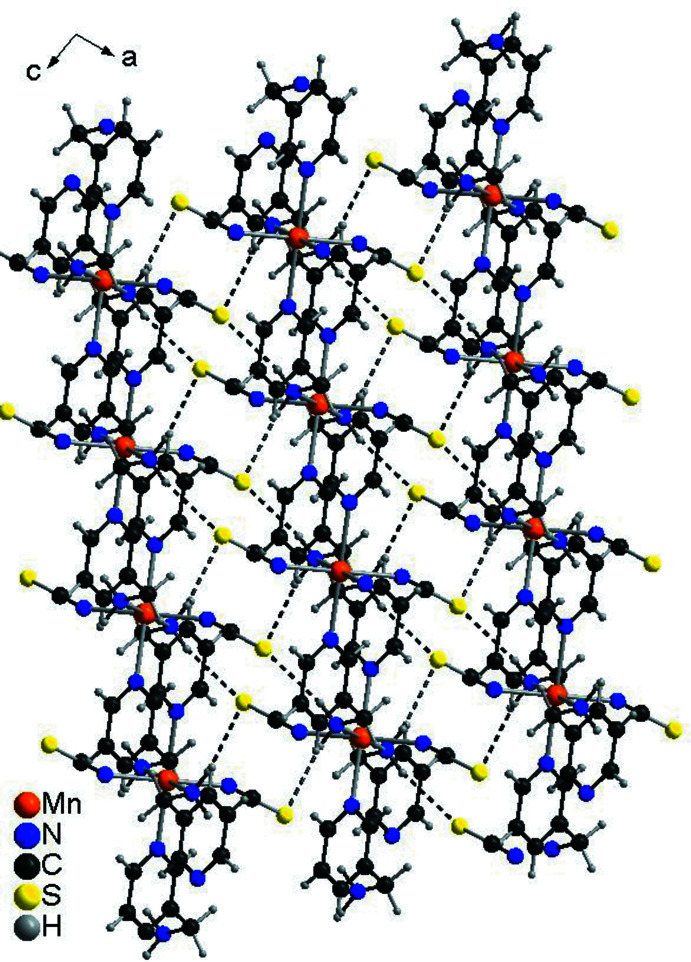
Crystal structure of the title compound in a view along the crystallographic *b* axis. Inter­molecular N—H⋯S hydrogen bonds are shown as dashed lines.

The layers are linked into a three-dimensional network by inter­molecular N—H⋯S hydrogen bonds between the amino H atoms and the thio­cyanate S atoms (Fig. 3 ▸, Table 2 ▸). The N—H⋯S angles indicate a relatively strong inter­action and the thio­cyanate S atom acts as an acceptor for two of these hydrogen bonds. There is also a C—H⋯S inter­action but the bonding angle is far from linearity, which points to a weak inter­action (Table 2 ▸).

## Database survey   

In the Cambridge Structure Database (CSD, version 5.42, last update November 2020; Groom *et al.*, 2016 ▸) no Mn–3-(amino­meth­yl)pyridine compounds are reported but a few compounds based on Zn(NCS)_2_ and Cd(NCS)_2_ have been deposited. In all of the corresponding structures, the metal cations are octa­hedrally coordinated. This includes *M*(NCS)_2_[3-(amino­meth­yl)pyridine]_2_ (*M* = Cd, Zn; Neumann *et al.*, 2017 ▸; refcodes: QEKZEO and QEKYUD), which are isotypic to the title compound, as well as *M*(NCS)_2_[3-(amino­meth­yl)pyridine] (*M* = Cd, Zn; Neumann, *et al.* 2017 ▸; refcodes: QEKZIS and QEKZAK). In the latter Zn^II^ compound, dimers are observed in which two Zn^II^ cations are connected by two 3-(amino­meth­yl)pyridine ligands. In the Cd^II^ compound, the metal cations are linked by *μ*-1,3-bridging thio­cyanate anions into chains that are connected into layers by the 3-(amino­meth­yl)pyridine ligands. This compound is the only one that contains *μ*-1,3-bridging thio­cyanate anions and which shows a *cis*-*cis*-*trans* coordination of the metal cations. There is also one solvate with the composition Cd(NCS)_2_[3-(amino­meth­yl)pyridine]_2_-tris­[3-(amino­meth­yl)pyridine] rep­orted in the CSD (refcode: QEKYOX; Neumann *et al.*, 2017 ▸). Finally, Co(NCS)_2_(3-(amino­meth­yl)pyridine)_2_, which is isotypic to the title compound, is also reported (Krebs *et al.*, 2021 ▸).

## Synthesis and crystallization   

**Synthesis**

Mn(NCS)_2_ and 3-(amino­meth­yl)pyridine were purchased from Alfa Aesar and all chemicals were used without further purification. Single crystals were obtained by reacting 1 mmol of Mn(NCS)_2_ (175.1 mg) with 1 mmol of 3-(amino­meth­yl)pyridine (108.1 mg) in 4 ml of ethanol. After approximately one week, light-brown crystals were obtained, which were suitable for single crystal X-ray analysis. For the synthesis of crystalline powders, the same amounts of reactants were stirred in 2 ml of ethanol for 1 d and the precipitate was filtered off and dried in air.

Elemental analysis calculated for C_14_H_16_N_6_MnS_2_: C 43.41%, H 4.16%, N 21.69%, S 16.55%; found: C 43.32%, H 4.11%, N 21.56, S 16.31%. IR: *ν* = 2971 (*m*), 2941 (*w*), 2928 (*w*), 2887 (*s*), 2875 (*w*), 2067 (*s*), 2023 (*m*), 1962(*vw*), 1861 (*vw*), 1595 (*m*), 1583 (*w*), 1480 (*m*), 1447 (*m*), 1426 (*m*), 1379 (*w*), 1361 (*w*), 1332 (*w*), 1274 (*wv*), 1244 (*w*), 1229 (*w*), 1189 (*m*), 1148 (*w*), 1124 (*m*), 1089 (*m*), 1048 (*vs*), 984 (*s*), 961 (*m*), 943 (*w*), 931 (*m*), 879 (*m*), 846 (*w*), 824 (*w*), 802 (*m*), 783 (*m*), 712 (*s*), 645 (*s*), 620 (*m*), 539 (*s*) cm^−1^.

**Experimental details**

The elemental analysis was performed using a EURO EA elemental analyzer fabricated by EURO VECTOR Instruments. The IR spectrum was measured using an ATI Mattson Genesis Series FTIR spectrometer, control software: *WINFIRST*, from ATI Mattson. The PXRD measurement was performed with Cu *K*α_1_ radiation (λ = 1.540598 Å) using a Stoe Transmission Powder Diffraction System (STADI P) that is equipped with a MYTHEN 1K detector and a Johansson-type Ge(111) monochromator. Thermogravimetry and differential thermoanalysis (TG–DTA) measurements were performed in a dynamic nitro­gen atmosphere in Al_2_O_3_ crucibles using a STA-PT 1000 thermobalance from Linseis. The instrument was calibrated using standard reference materials.

## Refinement   

Crystal data, data collection and structure refinement details are summarized in Table 3 ▸. All H atoms were located in a difference-Fourier map but were positioned with idealized geometry (N—H = 0.91 Å, C—H = 0.95–0.99 Å) and were refined in a riding model with *U*_iso_(H) = 1.2*U*_eq_(C) or 1.5*U*_eq_(C) for amino H atoms.

## Supplementary Material

Crystal structure: contains datablock(s) I. DOI: 10.1107/S2056989021006733/wm5613sup1.cif

Structure factors: contains datablock(s) I. DOI: 10.1107/S2056989021006733/wm5613Isup2.hkl

Figure S1. Experimental (top) and calculated X-ray powder pattern (bottom) of the title compound. For the calculation of the powder pattern the lattice parameters obtained from a Pawley fit of a powder pattern measured at room temperature were used. DOI: 10.1107/S2056989021006733/wm5613sup3.png

Figure S2. IR spectra of the title compound. The value of the CN stretching vibration of the thiocyanat anions is given. DOI: 10.1107/S2056989021006733/wm5613sup4.png

Figure S3. DTG (top), TG (middle) and DTA curve (bottom) of the title compound measured with 1K/min in nitrogen atmosphere. DOI: 10.1107/S2056989021006733/wm5613sup5.png

CCDC reference: 2092830

Additional supporting information:  crystallographic information; 3D view; checkCIF report

## Figures and Tables

**Table 1:** Selected geometric parameters (Å, °)

Mn1—N1	2.1955 (15)	Mn1—N11	2.3154 (14)
Mn1—N12^i^	2.2901 (14)		
			
N1—Mn1—N1^ii^	180.0	N1^ii^—Mn1—N11	90.82 (5)
N1—Mn1—N12^i^	91.17 (6)	N12^i^—Mn1—N11	89.52 (5)
N1—Mn1—N12^iii^	88.83 (6)	N12^iii^—Mn1—N11	90.48 (5)
N1—Mn1—N11	89.18 (5)	N1—Mn1—N11^ii^	90.82 (5)

Symmetry codes: (i) x-{\script{1\over 2}}, -y+{\script{3\over 2}}, z-{\script{1\over 2}}; (ii) -x, -y+1, -z; (iii) -x+{\script{1\over 2}}, y-{\script{1\over 2}}, -z+{\script{1\over 2}}.

**Table 2:** Hydrogen-bond geometry (Å, °)

*D*—H⋯*A*	*D*—H	H⋯*A*	*D*⋯*A*	*D*—H⋯*A*
C12—H12⋯S1^iii^	0.95	2.99	3.7264 (19)	136
N12—H12*A*⋯S1^iv^	0.91	2.81	3.7016 (16)	166
N12—H12*B*⋯S1^v^	0.91	2.66	3.5224 (15)	159

Symmetry codes: (iii) -x+{\script{1\over 2}}, y-{\script{1\over 2}}, -z+{\script{1\over 2}}; (iv) x, y, z+1; (v) x-{\script{1\over 2}}, -y+{\script{3\over 2}}, z+{\script{1\over 2}}.

**Table 3:** Experimental details

Crystal data	
Chemical formula	[Mn(NCS)_2_(C_6_H_8_N_2_S)_2_]
*M*_r_	387.39
Crystal system, space group	Monoclinic, *P*2_1_/*n*
Temperature (K)	200
*a*, *b*, *c* (Å)	8.2157 (3), 12.2356 (5), 8.9601 (3)
β (°)	99.736 (3)
*V* (Å^3^)	887.73 (6)
*Z*	2
Radiation type	Mo *K*α
μ (mm^−1^)	0.99
Crystal size (mm)	0.03 × 0.03 × 0.01

Data collection	
Diffractometer	Stoe *IPDS2*
Absorption correction	Numerical (*X-SHAPE* and *X-RED* 32; Stoe, 2002 ▸)
*T*_min_, *T*_max_	0.856, 0.980
No. of measured, independent and observed [*I* > 2σ(*I*)] reflections	12559, 1939, 1755
*R*_int_	0.040
(sin θ/λ)_max_ (Å^−1^)	0.639

Refinement	
*R*[*F* ^2^ > 2σ(*F* ^2^)], *wR*(*F* ^2^), *S*	0.031, 0.073, 1.14
No. of reflections	1939
No. of parameters	106
H-atom treatment	H-atom parameters constrained
Δρ_max_, Δρ_min_ (e Å^−3^)	0.27, −0.22

Computer programs: *X-AREA* (Stoe, 2002 ▸), *SHELXS97* (Sheldrick, 2008 ▸), *SHELXL2016/6* (Sheldrick, 2015 ▸), *DIAMOND* (Brandenburg & Putz, 1999 ▸) and *publCIF* (Westrip, 2010 ▸).

## supplementary crystallographic information

### Crystal data

**Table:**

[Mn(NCS)_2_(C_6_H_8_N_2_S)_2_]	*F*(000) = 398
*M**_r_* = 387.39	*D*_x_ = 1.449 Mg m^−^^3^
Monoclinic, *P*2_1_/*n*	Mo *K*α radiation, λ = 0.71073 Å
*a* = 8.2157 (3) Å	Cell parameters from 12559 reflections
*b* = 12.2356 (5) Å	θ = 2.8–27.0°
*c* = 8.9601 (3) Å	µ = 0.99 mm^−^^1^
β = 99.736 (3)°	*T* = 200 K
*V* = 887.73 (6) Å^3^	Block, light-brown
*Z* = 2	0.03 × 0.03 × 0.01 mm

### Data collection

**Table:**

Stoe IPDS-2 diffractometer	1755 reflections with *I* > 2σ(*I*)
ω scans	*R*_int_ = 0.040
Absorption correction: numerical (X-Shape and X-Red 32; Stoe, 2002)	θ_max_ = 27.0°, θ_min_ = 2.9°
*T*_min_ = 0.856, *T*_max_ = 0.980	*h* = −10→10
12559 measured reflections	*k* = −15→15
1939 independent reflections	*l* = −11→11

### Refinement

**Table:**

Refinement on *F*^2^	0 restraints
Least-squares matrix: full	Hydrogen site location: inferred from neighbouring sites
*R*[*F*^2^ > 2σ(*F*^2^)] = 0.031	H-atom parameters constrained
*wR*(*F*^2^) = 0.073	*w* = 1/[σ^2^(*F*_o_^2^) + (0.0359*P*)^2^ + 0.2401*P*] where *P* = (*F*_o_^2^ + 2*F*_c_^2^)/3
*S* = 1.14	(Δ/σ)_max_ = 0.001
1939 reflections	Δρ_max_ = 0.27 e Å^−^^3^
106 parameters	Δρ_min_ = −0.22 e Å^−^^3^

### Special details

**Table:**

Geometry. All esds (except the esd in the dihedral angle between two l.s. planes) are estimated using the full covariance matrix. The cell esds are taken into account individually in the estimation of esds in distances, angles and torsion angles; correlations between esds in cell parameters are only used when they are defined by crystal symmetry. An approximate (isotropic) treatment of cell esds is used for estimating esds involving l.s. planes.

### Fractional atomic coordinates and isotropic or equivalent isotropic displacement parameters (Å^2^)

**Table:**

	*x*	*y*	*z*	*U*_iso_*/*U*_eq_	
Mn1	0.000000	0.500000	0.000000	0.02622 (11)	
N1	0.20857 (19)	0.56497 (13)	−0.09665 (18)	0.0358 (3)	
C1	0.3146 (2)	0.62691 (14)	−0.10034 (19)	0.0313 (4)	
S1	0.46152 (6)	0.71704 (4)	−0.10505 (6)	0.04157 (14)	
N11	0.14907 (18)	0.52888 (12)	0.24007 (16)	0.0306 (3)	
C11	0.1329 (2)	0.46441 (15)	0.3576 (2)	0.0333 (4)	
H11	0.057240	0.405168	0.340862	0.040*	
C12	0.2207 (2)	0.47971 (15)	0.5022 (2)	0.0350 (4)	
H12	0.206448	0.431458	0.582080	0.042*	
C13	0.3292 (2)	0.56627 (15)	0.5281 (2)	0.0339 (4)	
H13	0.391568	0.577949	0.626157	0.041*	
C14	0.3466 (2)	0.63626 (14)	0.40992 (19)	0.0295 (3)	
C15	0.2556 (2)	0.61299 (14)	0.2685 (2)	0.0316 (3)	
H15	0.269345	0.659337	0.186448	0.038*	
C16	0.4577 (2)	0.73525 (14)	0.4322 (2)	0.0340 (4)	
H16A	0.564073	0.714074	0.494763	0.041*	
H16B	0.480824	0.759048	0.332262	0.041*	
N12	0.38812 (18)	0.82892 (11)	0.50551 (17)	0.0319 (3)	
H12A	0.391635	0.810986	0.604571	0.038*	
H12B	0.279589	0.834201	0.463345	0.038*	

### Atomic displacement parameters (Å^2^)

**Table:**

	*U*^11^	*U*^22^	*U*^33^	*U*^12^	*U*^13^	*U*^23^
Mn1	0.02752 (18)	0.02188 (18)	0.02836 (19)	−0.00181 (13)	0.00218 (13)	0.00086 (13)
N1	0.0343 (8)	0.0370 (8)	0.0371 (8)	−0.0054 (6)	0.0086 (6)	0.0015 (7)
C1	0.0330 (8)	0.0321 (9)	0.0291 (8)	0.0047 (7)	0.0060 (7)	−0.0012 (7)
S1	0.0355 (2)	0.0368 (3)	0.0526 (3)	−0.00815 (19)	0.0079 (2)	−0.0034 (2)
N11	0.0327 (7)	0.0273 (7)	0.0305 (7)	−0.0022 (5)	0.0019 (6)	−0.0013 (5)
C11	0.0362 (9)	0.0276 (8)	0.0361 (9)	−0.0047 (7)	0.0063 (7)	−0.0024 (7)
C12	0.0424 (9)	0.0305 (9)	0.0326 (9)	−0.0020 (7)	0.0074 (7)	0.0022 (7)
C13	0.0396 (9)	0.0322 (9)	0.0291 (8)	0.0000 (7)	0.0039 (7)	−0.0021 (7)
C14	0.0302 (8)	0.0250 (8)	0.0333 (8)	0.0008 (6)	0.0054 (7)	−0.0033 (6)
C15	0.0359 (8)	0.0269 (8)	0.0313 (8)	−0.0010 (7)	0.0038 (7)	0.0012 (6)
C16	0.0348 (9)	0.0281 (9)	0.0389 (9)	−0.0027 (7)	0.0061 (7)	−0.0043 (7)
N12	0.0334 (7)	0.0253 (7)	0.0368 (8)	−0.0023 (6)	0.0048 (6)	−0.0017 (6)

### Geometric parameters (Å, º)

**Table:**

Mn1—N1	2.1955 (15)	C12—C13	1.378 (3)
Mn1—N1^i^	2.1955 (15)	C12—H12	0.9500
Mn1—N12^ii^	2.2901 (14)	C13—C14	1.388 (2)
Mn1—N12^iii^	2.2901 (14)	C13—H13	0.9500
Mn1—N11	2.3154 (14)	C14—C15	1.388 (2)
Mn1—N11^i^	2.3154 (14)	C14—C16	1.509 (2)
N1—C1	1.159 (2)	C15—H15	0.9500
C1—S1	1.6406 (18)	C16—N12	1.483 (2)
N11—C11	1.340 (2)	C16—H16A	0.9900
N11—C15	1.347 (2)	C16—H16B	0.9900
C11—C12	1.385 (3)	N12—H12A	0.9100
C11—H11	0.9500	N12—H12B	0.9100
			
N1—Mn1—N1^i^	180.0	C13—C12—H12	120.6
N1—Mn1—N12^ii^	91.17 (6)	C11—C12—H12	120.6
N1^i^—Mn1—N12^ii^	88.83 (6)	C12—C13—C14	119.54 (17)
N1—Mn1—N12^iii^	88.83 (6)	C12—C13—H13	120.2
N1^i^—Mn1—N12^iii^	91.17 (6)	C14—C13—H13	120.2
N12^ii^—Mn1—N12^iii^	180.0	C15—C14—C13	117.45 (16)
N1—Mn1—N11	89.18 (5)	C15—C14—C16	120.37 (16)
N1^i^—Mn1—N11	90.82 (5)	C13—C14—C16	122.17 (16)
N12^ii^—Mn1—N11	89.52 (5)	N11—C15—C14	124.18 (16)
N12^iii^—Mn1—N11	90.48 (5)	N11—C15—H15	117.9
N1—Mn1—N11^i^	90.82 (5)	C14—C15—H15	117.9
N1^i^—Mn1—N11^i^	89.18 (5)	N12—C16—C14	114.17 (14)
N12^ii^—Mn1—N11^i^	90.48 (5)	N12—C16—H16A	108.7
N12^iii^—Mn1—N11^i^	89.52 (5)	C14—C16—H16A	108.7
N11—Mn1—N11^i^	180.0	N12—C16—H16B	108.7
C1—N1—Mn1	152.67 (14)	C14—C16—H16B	108.7
N1—C1—S1	178.57 (16)	H16A—C16—H16B	107.6
C11—N11—C15	116.67 (15)	C16—N12—Mn1^iv^	120.72 (11)
C11—N11—Mn1	122.20 (11)	C16—N12—H12A	107.1
C15—N11—Mn1	121.12 (11)	Mn1^iv^—N12—H12A	107.1
N11—C11—C12	123.38 (16)	C16—N12—H12B	107.1
N11—C11—H11	118.3	Mn1^iv^—N12—H12B	107.1
C12—C11—H11	118.3	H12A—N12—H12B	106.8
C13—C12—C11	118.75 (17)		

Symmetry codes: (i) −*x*, −*y*+1, −*z*; (ii) *x*−1/2, −*y*+3/2, *z*−1/2; (iii) −*x*+1/2, *y*−1/2, −*z*+1/2; (iv) −*x*+1/2, *y*+1/2, −*z*+1/2.

### Hydrogen-bond geometry (Å, º)

**Table:**

*D*—H···*A*	*D*—H	H···*A*	*D*···*A*	*D*—H···*A*
C12—H12···S1^iii^	0.95	2.99	3.7264 (19)	136
N12—H12*A*···S1^v^	0.91	2.81	3.7016 (16)	166
N12—H12*B*···S1^vi^	0.91	2.66	3.5224 (15)	159

Symmetry codes: (iii) −*x*+1/2, *y*−1/2, −*z*+1/2; (v) *x*, *y*, *z*+1; (vi) *x*−1/2, −*y*+3/2, *z*+1/2.

## References

[bb1] Bassey, E. N., Paddison, J. A. M., Keyzer, E. N., Lee, J., Manuel, P., da Silva, I., Dutton, S. E., Grey, C. P. & Cliffe, M. J. (2020). *Inorg. Chem.* **59**, 11627–11639. 10.1021/acs.inorgchem.0c01478 32799496

[bb2] Böhme, M., Jochim, A., Rams, M., Lohmiller, T., Suckert, S., Schnegg, A., Plass, W. & Näther, C. (2020). *Inorg. Chem.* **59**, 5325–5338. 10.1021/acs.inorgchem.9b03357 32091883

[bb3] Brandenburg, K. & Putz, H. (1999). *DIAMOND*. Crystal Impact GbR, Bonn, Germany.

[bb4] Groom, C. R., Bruno, I. J., Lightfoot, M. P. & Ward, S. C. (2016). *Acta Cryst.* B**72**, 171–179. 10.1107/S2052520616003954 PMC4822653 27048719

[bb5] Jin, Y., Che, Y. X. & Zheng, J. M. (2007). *J. Coord. Chem.* **60**, 2067–2074.

[bb6] Jochim, A., Lohmiller, T., Rams, M., Böhme, M., Ceglarska, M., Schnegg, A., Plass, W. & Näther, C. (2020). *Inorg. Chem.* **59**, 8971–8982. 10.1021/acs.inorgchem.0c00815 32551545

[bb7] Jochim, A., Rams, M., Neumann, T., Wellm, C., Reinsch, H., Wójtowicz, G. M. & Näther, C. (2018). *Eur. J. Inorg. Chem.* pp. 4779–4789.

[bb8] Krebs, C., Jess, I. & Näther, C. (2021). *Acta Cryst.* E**77**, 428–432. 10.1107/S2056989021003005 PMC8025872 33936771

[bb9] Małecki, J. G., Machura, B., Świtlicka, A., Groń, T. & Bałanda, M. (2011). *Polyhedron*, **30**, 746–753.

[bb10] Mautner, F. A., Scherzer, M., Berger, C., Fischer, R. C., Vicente, R. & Massoud, S. S. (2015). *Polyhedron*, **85**, 20–26.

[bb11] Mautner, F. A., Traber, M., Fischer, R. C., Torvisco, A., Reichmann, K., Speed, S., Vicente, R. & Massoud, S. S. (2018). *Polyhedron*, **154**, 436–442.

[bb12] Mekuimemba, C. D., Conan, F., Mota, A. J., Palacios, M. A., Colacio, E. & Triki, S. (2018). *Inorg. Chem.* **57**, 2184–2192. 10.1021/acs.inorgchem.7b03082 29420016

[bb13] Mousavi, M., Duhayon, C., Bretosh, K., Béreau, V. & Sutter, J. P. (2020). *Inorg. Chem.* **59**, 7603–7613. 10.1021/acs.inorgchem.0c00459 32412746

[bb14] Neumann, T., Germann, L. S., Moudrakovski, I., Dinnebier, R. E., dos Santos Cunha, C., Terraschke, H. & Näther, C. (2017). *Z. Anorg. Allg. Chem.* **643**, 1904–1912.

[bb15] Neumann, T., Rams, M., Tomkowicz, Z., Jess, I. & Näther, C. (2019). *Chem. Commun.* **55**, 2652–2655. 10.1039/c8cc09392j 30742155

[bb16] Palion-Gazda, J., Machura, B., Lloret, F. & Julve, M. (2015). *Cryst. Growth Des.* **15**, 2380–2388.

[bb17] Prananto, Y. P., Urbatsch, A., Moubaraki, B., Murray, K. S., Turner, D. R., Deacon, G. B. & Batten, S. R. (2017). *Aust. J. Chem.* **70**, 516–528.

[bb18] Rams, M., Jochim, A., Böhme, M., Lohmiller, T., Ceglarska, M., Rams, M. M., Schnegg, A., Plass, W. & Näther, C. (2020). *Chem. Eur. J.* **26**, 2837–2851. 10.1002/chem.201903924 PMC7078958 31702081

[bb19] Rams, M., Tomkowicz, Z., Böhme, M., Plass, W., Suckert, S., Werner, J., Jess, I. & Näther, C. (2017). *Phys. Chem. Chem. Phys.* **19**, 3232–3243. 10.1039/c6cp08193b 28083584

[bb20] Robinson, K., Gibbs, G. V. & Ribbe, P. H. (1971). *Science*, **172**, 567–570. 10.1126/science.172.3983.567 17802221

[bb21] Sheldrick, G. M. (2008). *Acta Cryst.* A**64**, 112–122. 10.1107/S0108767307043930 18156677

[bb22] Sheldrick, G. M. (2015). *Acta Cryst.* C**71**, 3–8.

[bb23] Stoe (2002). *X-AREA* and *X-RED32.* Stoe & Cie, Darmstadt, Germany.

[bb24] Suckert, S., Rams, M., Böhme, M., Germann, L. S., Dinnebier, R. E., Plass, W., Werner, J. & Näther, C. (2016). *Dalton Trans.* **45**, 18190–18201. 10.1039/c6dt03752f 27796392

[bb25] Suckert, S., Rams, M., Germann, L. S., Cegiełka, D. M., Dinnebier, R. E. & Näther, C. (2017). *Cryst. Growth Des.* **17**, 3997–4005.

[bb26] Wei, R. & Luo, F. (2010). *J. Coord. Chem.* **63**, 610–616.

[bb27] Werner, S., Tomkowicz, Z., Rams, M., Ebbinghaus, S. G., Neumann, T. & Näther, C. (2015). *Dalton Trans.* **44**, 14149–14158. 10.1039/c5dt01898f 26182402

[bb28] Westrip, S. P. (2010). *J. Appl. Cryst.* **43**, 920–925.

[bb29] Wöhlert, S., Runčevski, T., Dinnebier, R. E., Ebbinghaus, S. G. & Näther, C. (2014). *Cryst. Growth Des.* **14**, 1902–1913.

