# Supplementary figures and images for: Synthesis, crystal structure and thermal properties of poly[bis­[μ-3-(amino­meth­yl)pyridine-κ2 N:N′]bis(thio­cyanato-κN)manganese(II)]
Source: Acta Crystallogr E Crystallogr Commun. 2021 Jul 2;77(Pt 8):765–9. doi: 10.1107/S2056989021006733 (PMC8340975; doi:10.1107/S2056989021006733)

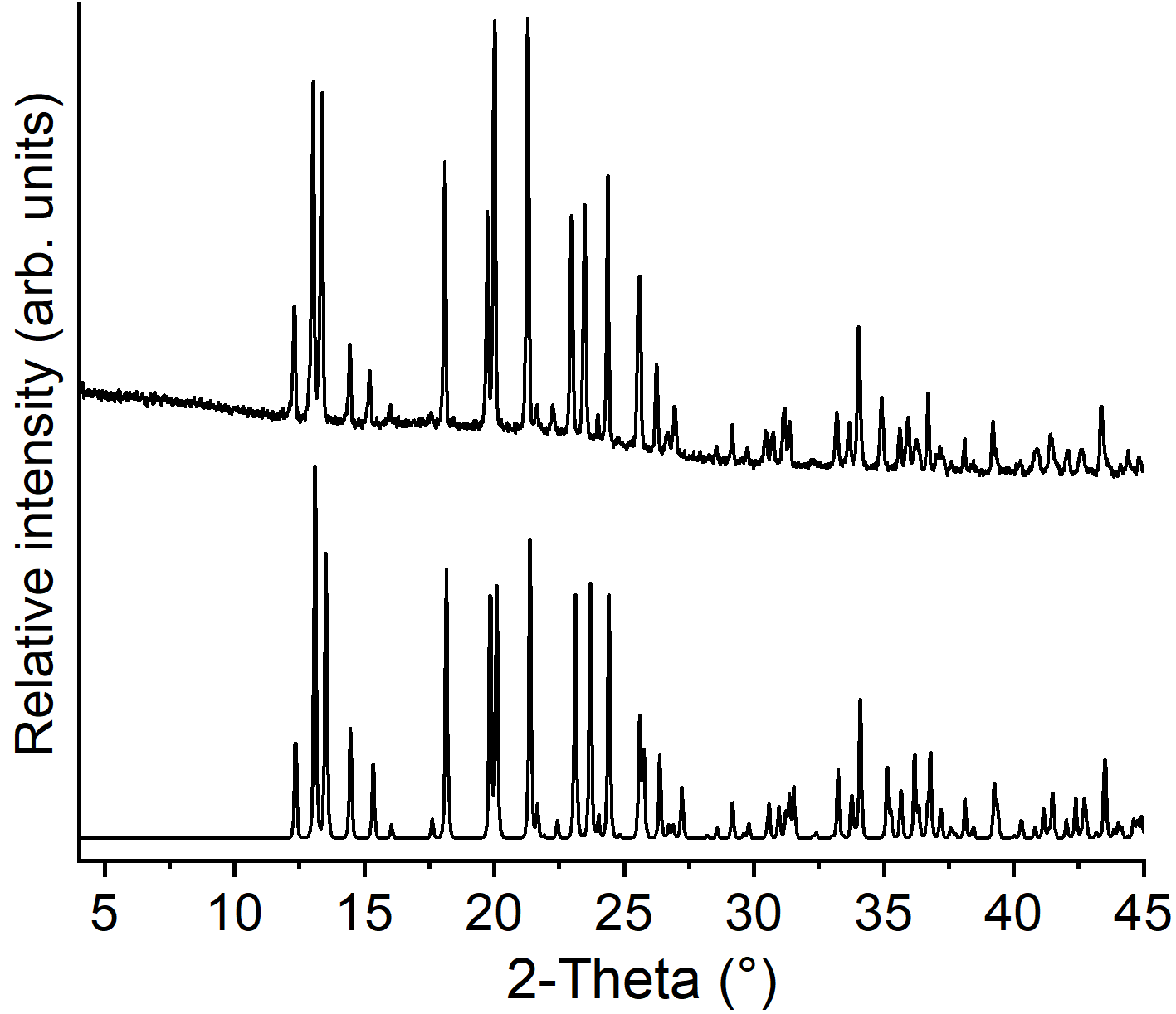

Supplement: Supplementary file 3 [file e-77-00765-sup3.png]

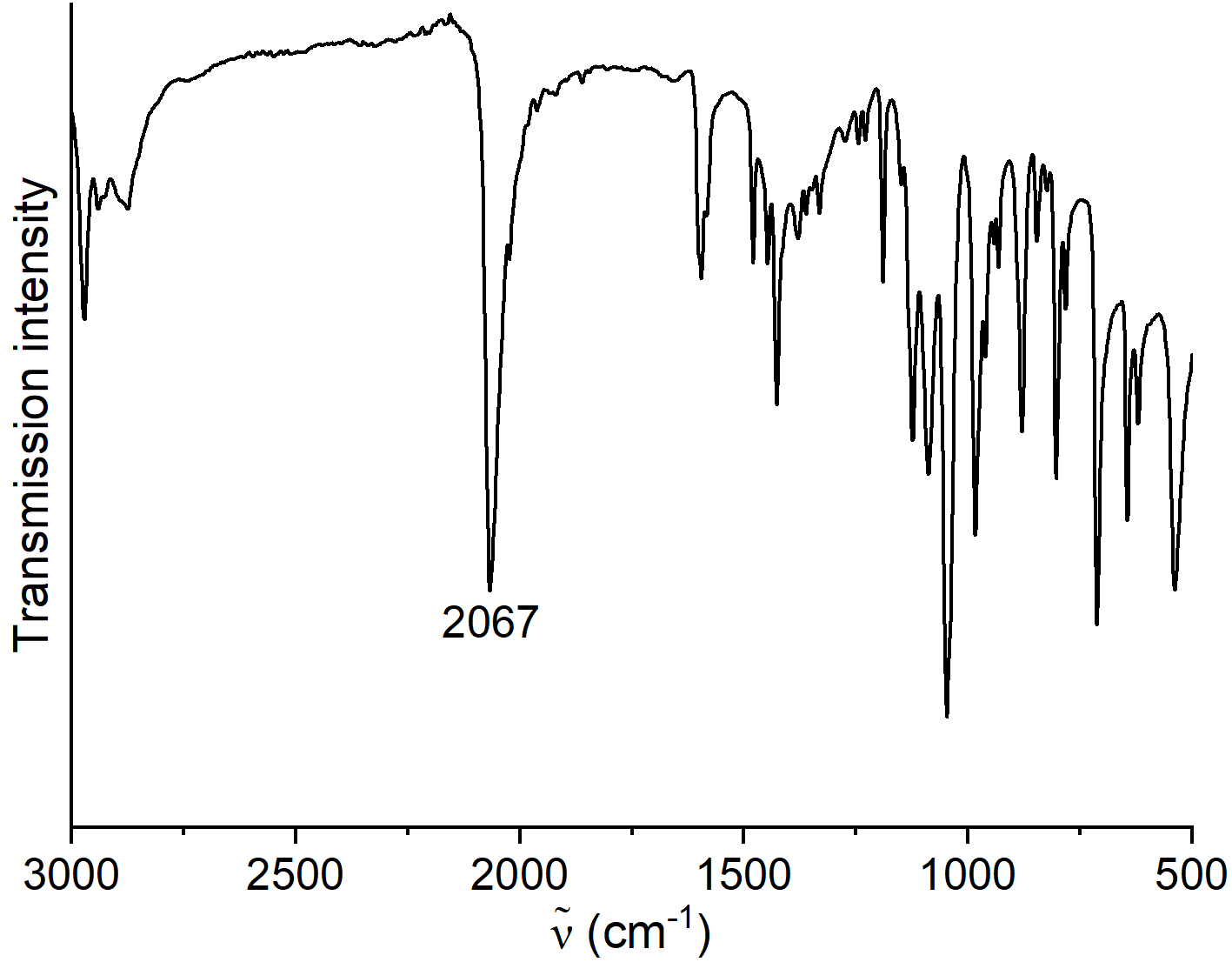

Supplement: Supplementary file 4 [file e-77-00765-sup4.png]

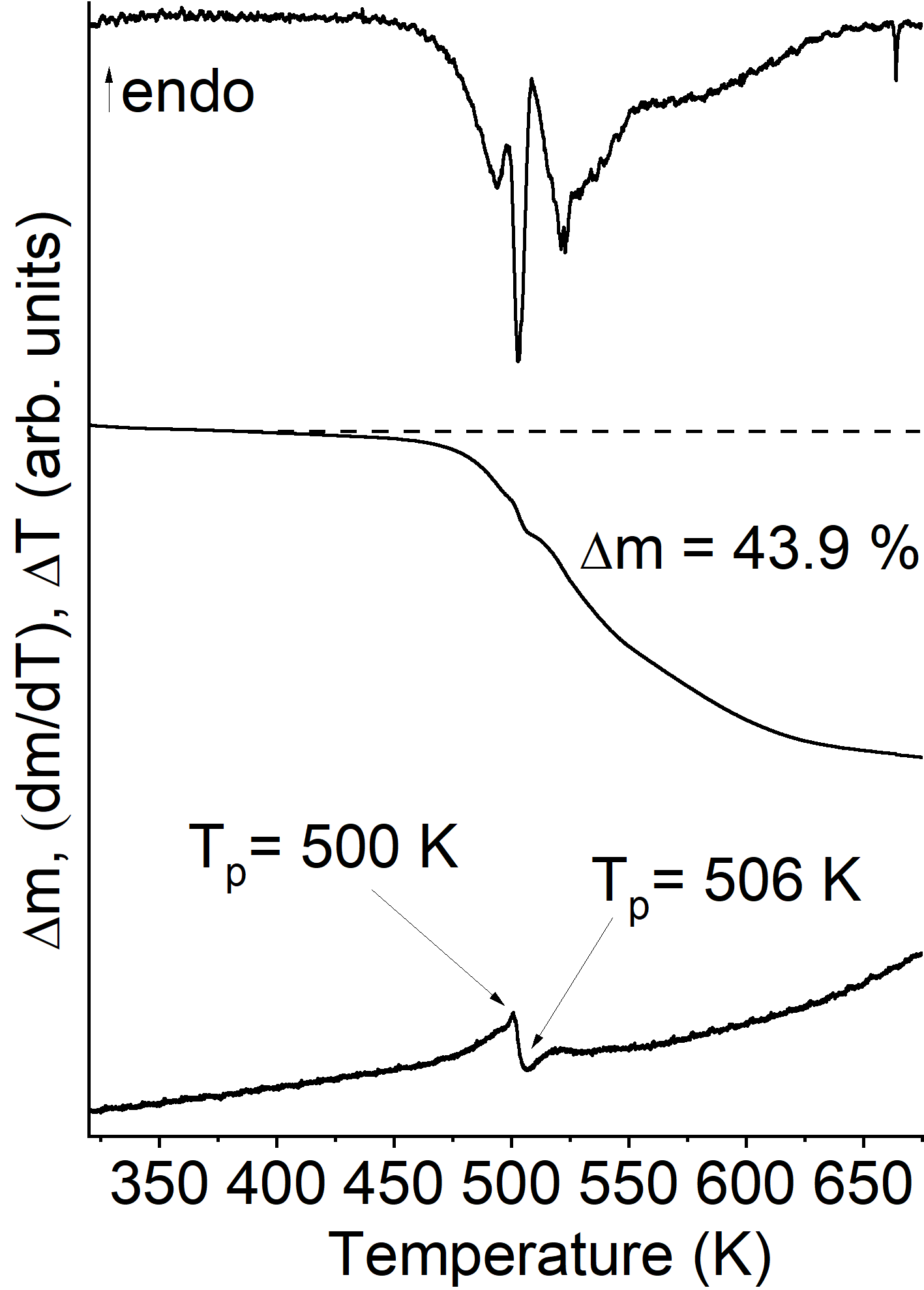

Supplement: Supplementary file 5 [file e-77-00765-sup5.png]
